# Evolutionary and Antigenic Profiling of the Tendentious D614G Mutation of SARS-CoV-2 in Gujarat, India

**DOI:** 10.3389/fgene.2021.764927

**Published:** 2021-11-11

**Authors:** Jay Nimavat, Chandrashekar Mootapally, Neelam M. Nathani, Devyani Dave, Mukesh N. Kher, Mayur S. Mahajan, Chaitanya G. Joshi, Vaibhav D. Bhatt

**Affiliations:** ^1^ Department of Pharmaceutical Sciences, Saurashtra University, Rajkot, India; ^2^ School of Applied Sciences and Technology (GTU-SAST), Gujarat Technological University, Ahmedabad, India; ^3^ L. M. College of Pharmacy, Ahmedabad, India; ^4^ Atal Incubation Centre, Gujarat Technological University, Ahmedabad, India; ^5^ Gujarat Biotechnology Research Centre, Department of Science and Technology, Gandhinagar, India

**Keywords:** antigenic propensity, clades, D614G, SARS-CoV-2, spike glycoprotein

## Abstract

Humankind has suffered many pandemics in history including measles, SARS, MERS, Ebola, and recently the novel Coronavirus disease caused by SARS-CoV-2. As of September 2021, it has affected over 200 million people and caused over 4 million deaths. India is the second most affected country in the world. Up to this date, more than 38 Lakh viral genomes have been submitted to public repositories like GISAID and NCBI to analyze the virus phylogeny and mutations. Here, we analyzed 2349 genome sequences of SARS-CoV-2 submitted in GISAID by a single institute pertaining to infections from the Gujarat state to know their variants and phylogenetic distributions with a major focus on the spike protein. More than 93% of the genomes had one or more mutations in the spike glycoprotein. The D614G variant in spike protein is reported to have a very high frequency of >95% globally followed by the L452R and P681R, thus getting significant attention. The antigenic propensity of a small peptide of 29 residues from 597 to 625 of the spike protein variants having D614 and G614 showed that G614 has a little higher antigenic propensity. Thus, the D614G is the cause for higher viral antigenicity, however, it has not been reported to be effective to be causing more deaths.

## Introduction

In the last 2 decades, this is the third instance of a zoonotic coronavirus pandemic. Acute respiratory disease has previously been caused by SARS-CoV, 2002 (Drosten et al., 2003) and MERS-CoV, 2012 (Azhar et al., 2014a; Azhar et al., 2014b) in humans. The novel SARS-CoV-2 virus has recently triggered the coronavirus disease Covid-19. The viral infection began in Wuhan, China in December 2019 and soon became a global outbreak. In a very short span, it has caused significant effects on social and economic activities. Compared to other coronaviruses, this newly emerged SARS-CoV-2 is spreading rapidly, giving challenges to administrative and scientific communities. Influenza, severe respiratory, enteric and neurological complications, elevated white blood cells, and kidney failure are significant indicators of this viral infection. Mammals such as bats are the primary beta coronavirus reservoirs. Due to zoonotic contacts and viral genomic mutations, it is expected to have crossed the species barrier and infected humans. Previous studies indicate that zoonotic infections such as SARS-CoV was transmitted from bats and civets that first infected humans in 2002 (Ksiazek et al., 2003; Marra et al., 2003; Rota et al., 2003; Xu et al., 2004). SARS-CoV-2 is enveloped, contains positive sense ssRNA, and a genome size of 29–30 kb. It belongs to the coronaviridae family and subfamily beta-coronavirus. The family also comprises of MERS CoV which was originated from camel and later led to human transmission in Saudi Arabia (2012) (Azhar et al., 2014a; Chan et al., 2015; Sabir et al., 2016). These infections from bats are predicted to infect humans due to their change in genomic RNA sequence, especially in the spike glycoprotein region (Song et al., 2005; Menachery et al., 2016). Complete genomic sequences of SARS-CoV-2 isolated from infected patients belonging to different geographical locations allows the understanding of these variations and the corresponding influence on viral infecting potency.

SARS CoV-2 similar to SARS-CoV uses angiotensin-converting enzyme II (ACE2) as a receptor for host cell entry. It has spike glycoproteins on the surface, which has two functional domains, S1 and S2. It helps in host cell receptor binding and fusion of viral membrane with the cellular membrane (Harvey et al., 2021). SARS-CoV-2 spike protein has an ACE2 affinity 10 to 20 times greater than that of SARS-CoV spike protein (Walls et al., 2020; Wrapp et al., 2020). Both SARS-CoV and SARS-CoV-2 use CTD (C- terminal domain) of the S1 domain for receptor binding but SARS-CoV-2 binds more strongly than SARS-CoV. Coronaviruses use two different pathways for host cell entry, First, protease mediated cell surface pathway and second, the endosomal pathway. S protein is cleaved into an S1 subunit for receptor binding and an S2 subunit for membrane fusion by the host proteases. Several cellular proteases including furin, transmembrane protease serine 2 (TMPRSS2) and cathepsin (cat) B/L are important for priming SARS-CoV-2 spike protein to enhance ACE2 mediated viral entry (Hoffmann et al., 2020). Spike protein plays a vital role in the evolution of coronaviruses to escape the host immune system. Spike protein shows a higher amount of antigenicity, which is evident from the fact that convalescent plasma from SARS patients shows a high percentage of anti-S neutralizing antibodies (Liu et al., 2006; Wan et al., 2020). There are a lot of variations observed in spike protein sequence, a major variation in spike protein is a non-synonymous D614G mutation (d-Aspartate, G-Glycine) which has received special attention by several groups due to its dominance (Harvey et al., 2021).

Several studies have reported the phylogenomics of the variant and shown that it is leading to higher transmission, though no less influence on the death rate. Few studies have also shown that the variant has increased cellular entry efficacy to human cells compared to the wild type (Wan et al., 2020). In context to the same, here we assessed the antigenic propensity of the epitope encompassing the D614G mutation considering its high frequency and the segment being earlier reported as immune-dominant peptide in SARS-CoV (Wang et al., 2016; Kim et al., 2020). The region-wise analysis of the variant will provide information of this rapidly spreading variant for possible considerations in protective strategy development. Further, we also compiled the current major spike mutations in the Gujarat state in comparison with their global frequencies.

## Materials and Method

### SARS-CoV-2 Sequence Retrieval

A total of 2439 sequences of SARS-CoV-2 genome sequences corresponding to the Gujarat state were retrieved from GISAID (https://www.gisaid.org/) hCov-19 database and these sequences represented different districts of Gujarat state. The genomes were sequenced and submitted by Gujarat Biotechnology Research. The complete genome sequences of SARS-CoV-2 reference genome of the Wuhan isolate (GenBank code: MN908947.3) and Bat CoV-RaTG13 (MN996532.1) were retrieved in FASTA format from NCBI. All the retrieved sequences were subjected to BLAST.

### Mutation and Phylogenetic Analysis

Spike protein sequences retrieved from GISAID were aligned with reference spike protein sequences using Jalview version 2.11.1.0 (Waterhouse et al., 2009). Mutations were identified and listed using GISAID EpiCoV™ database (Elbe and Buckland-Merrett, 2017). Occurrence of D614G mutation over time was visualized using NextStrain platform (Hadfield et al., 2018) where data is enabled from the GISAID. NextStrain visualization analysis can process up to 3000 genomic sequences at a time. Therefore, for the primary global analysis, they subsample 120 genomes per admin division per month giving results in a more equitable way.

### Antigenic Propensity Analysis

A small part of sequence of spike glycoprotein S_597-625_ from sequences was analyzed using the method described earlier (Kolaskar and Tongaonkar, 1990) provided on an online server by UNIVERSIDAD COMPLUTENSE, MADRID. The interpretation was done as suggested: the average score for the whole protein was used as a cut-off for the then all residues to be considered as potentially antigenic.

## Results

### Sequence Similarity Studies of SARS-CoV-2 Genomes

A total of 2439 sequences of SARS-CoV-2 were retrieved from GISAID platform. Upon performing nucleotide BLAST of reference SARS-CoV-2 with SARS CoV, at the genomic level SARS-CoV-2 and SARS-CoV were observed to have 79.6% sequence similarity. Sequences retrieved from infected patients by SARS-Cov-2 from GISAID had sequence similarity of about 80% with SARS-CoV, 99.9% with SARS-CoV-2 reference sequence, and 96% with the Bat CoV RaTG13.

### Mutation Analysis of SARS-CoV-2 Genomes

**FIGURE 1:**
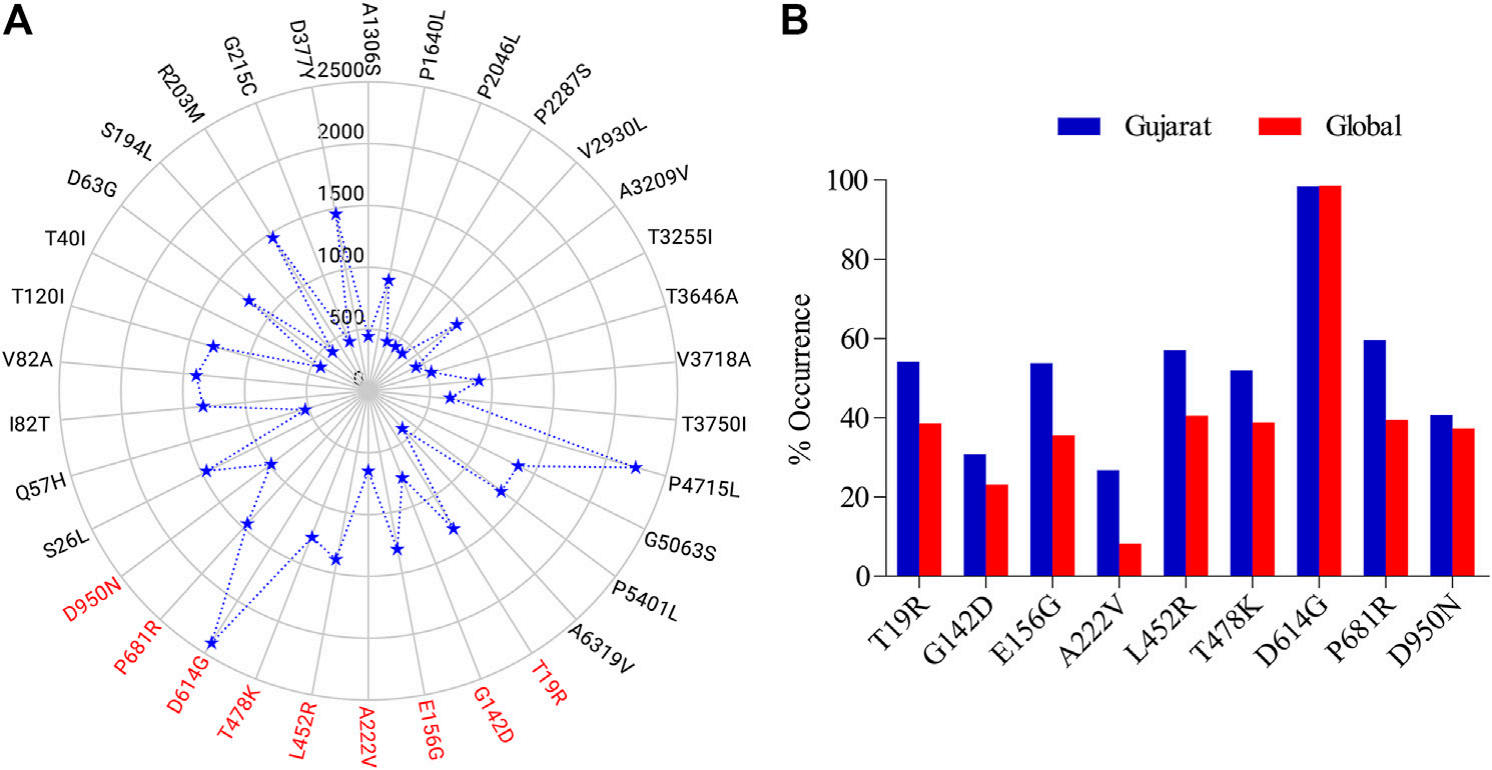
Non synonymous mutations as observed **(A)** in the analysed SARS-CoV-2 genomes (*n* = 2,439) from Gujarat region (submissions by GBRC) compared to the Wuhan SARS-CoV-2 reference, scale represents the number of genomes, those highlighted in red are specific to the spike glycoprotein, **(B)** in the spike glycoprotein of the sequences from Gujarat (*n* = 2,439) and globally (*n* = 3,897,179) in terms of their percent occurrence as on September, 2021.

Out of 2439 sequences studied, 2400 genomes had a common mutation and there were 9 mutations observed in spike protein that were present in at least 15% of the studied genomes (Figure 1). A total of 34 nonsynonymous mutations were observed, with the spike D614G having the highest occurrence in 2400 genomes followed by the nsp12 P4715L mutation observed in 2254 genomes (Figure 1A). Nine of the spike mutations had a percentage occurrence in the range of 25–99 in the studied genomes (Figure 1B). After D614G, P681R occurred in 1455 of the analyzed sequences.

**FIGURE 2:**
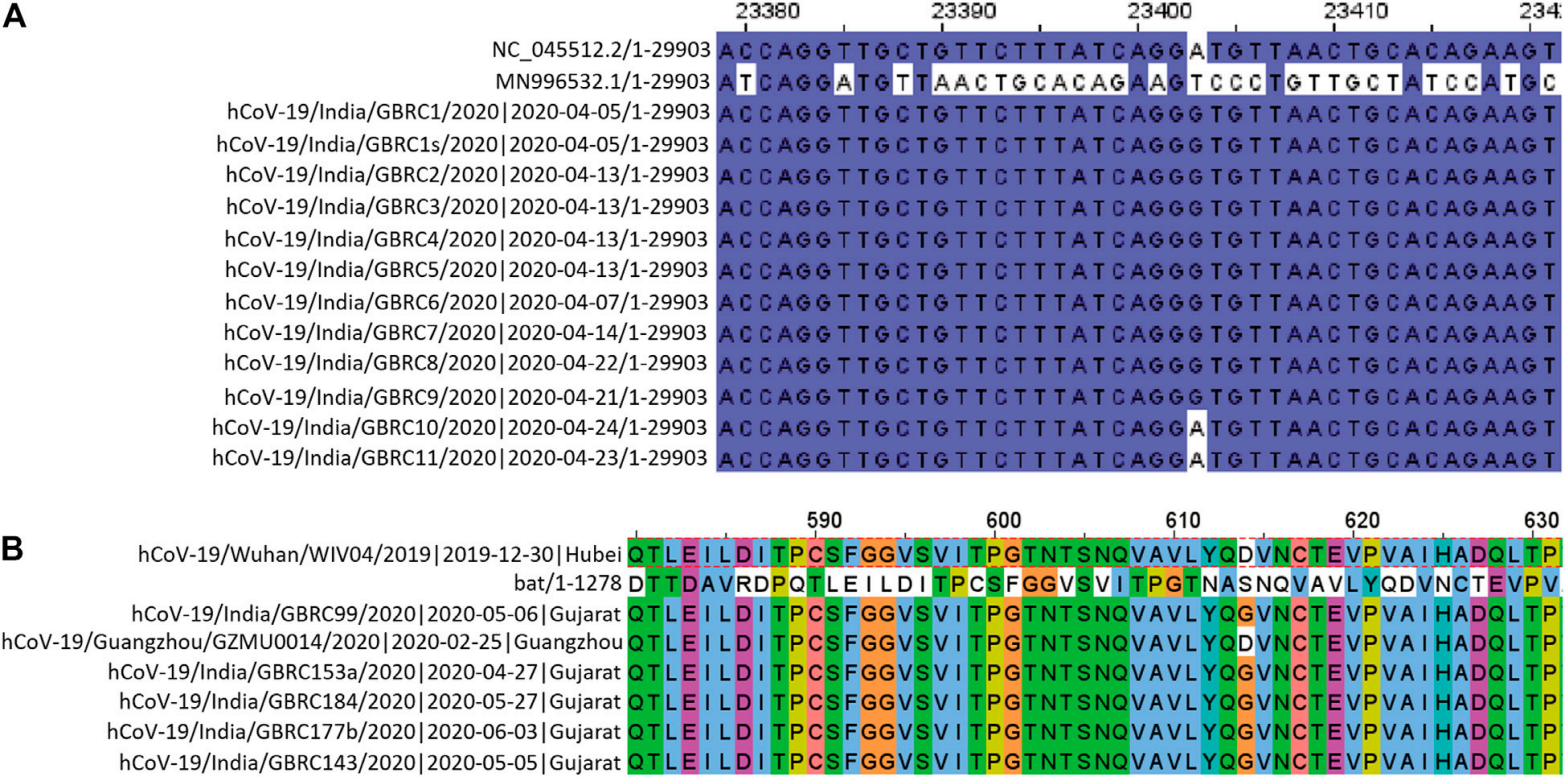
**(A)** Change in the nucleotide at position 23403. NC_045512.2/1-29903 and MN996532.1/1-29855 are SARS-CoV-2 reference sequence and Bat CoV RaTG13 reference sequence, respectively. **(B)** Amino acid sequence alignment of spike protein encompassing the D614G position.

All, except D614G, from the 9 non-synonymous mutations observed, had relatively more frequency in the Gujarat region compared to their global frequencies. While D614G had almost similar frequency with its global value. In the genomic sequence for spike glycoprotein, a single mutation, i.e., from A to G nucleotide was prevalent in the majority of genomes at the nucleotide position number 23403 (Figure 2). This change in the nucleotide causes change in amino acid while translating the gene, because of this aspartate is replaced by glycine in the protein sequence (Figure 2). Its frequency of occurrence has overall increased with the time. Phylogenomics of genomes based on the D and G variants is depicted at the time of initial data collection and the scenario down to the recent timeline (Supplementary Figure S1). Also, there is a clear difference in frequency as observed for D614G which was comparatively much higher compared to global during early phase up to July 2020 (Supplementary Figure S2) and now the frequencies are almost the same. Such difference currently is observed in the P681R mutation, which is the hallmark mutation of the Delta variant wherein the percentage occurrence is 20% higher in the Gujarat region compared to the global. The P681R has outraced other major mutations in spike proteins and is the second major mutation.

**FIGURE 3:**
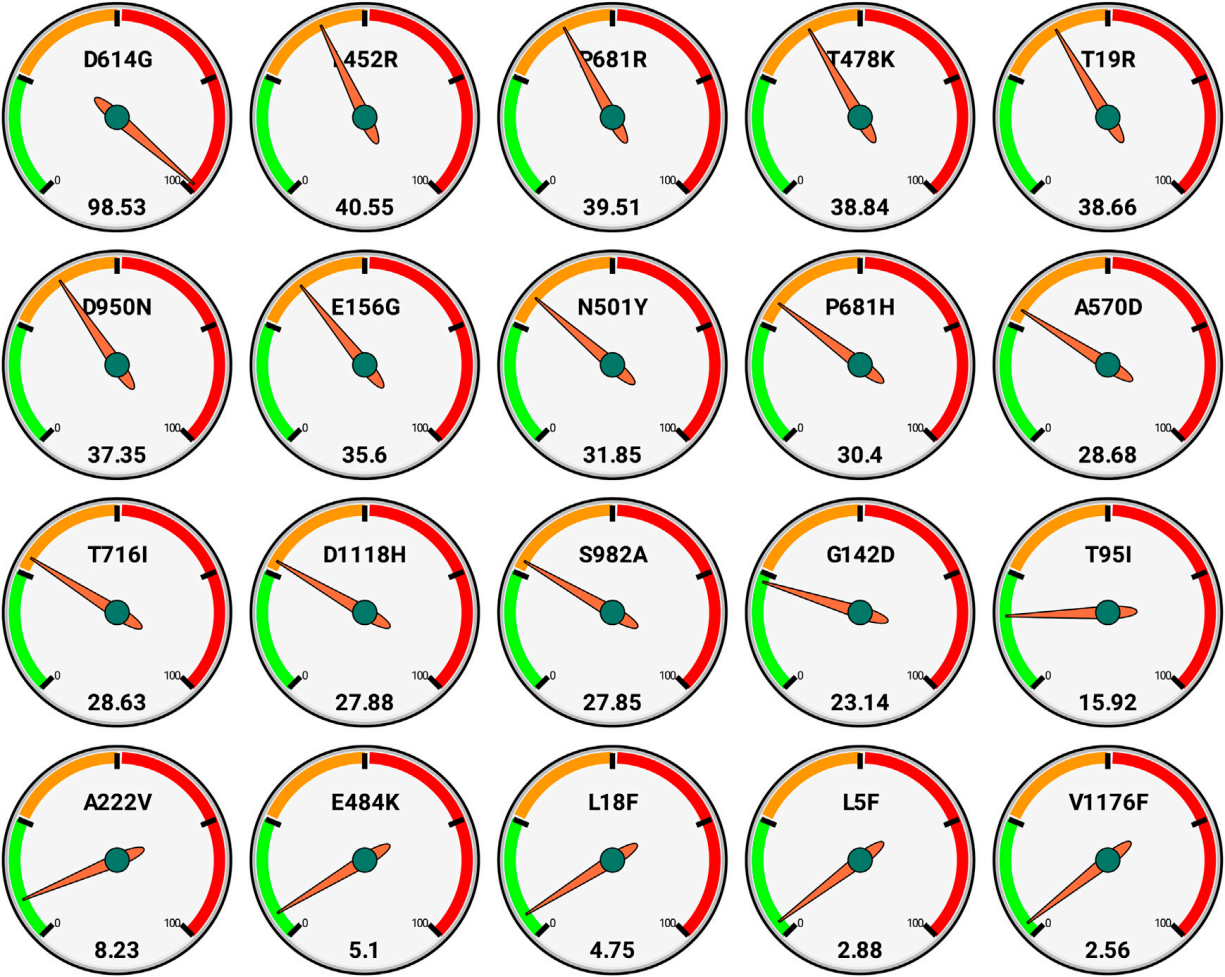
Global frequencies of top 20 non synonymous mutations in the spike protein of the SARS-CoV-2 genomes (*n* = 3,897,179) as available in GISAID as on date September 30, 2021. Each gauge has a frequency scale (0–100) divided quarterly and represents single mutation (top) along with its respective frequency (bottom).

We also report here the top 20 nonsynonymous mutations as per global scenario with their frequencies (Figure 3). These also reflect that D613G is the most spread, and further there are around 12 mutations that have >25% occurrence globally.

### Antigenicity of Peptides

**FIGURE 4:**
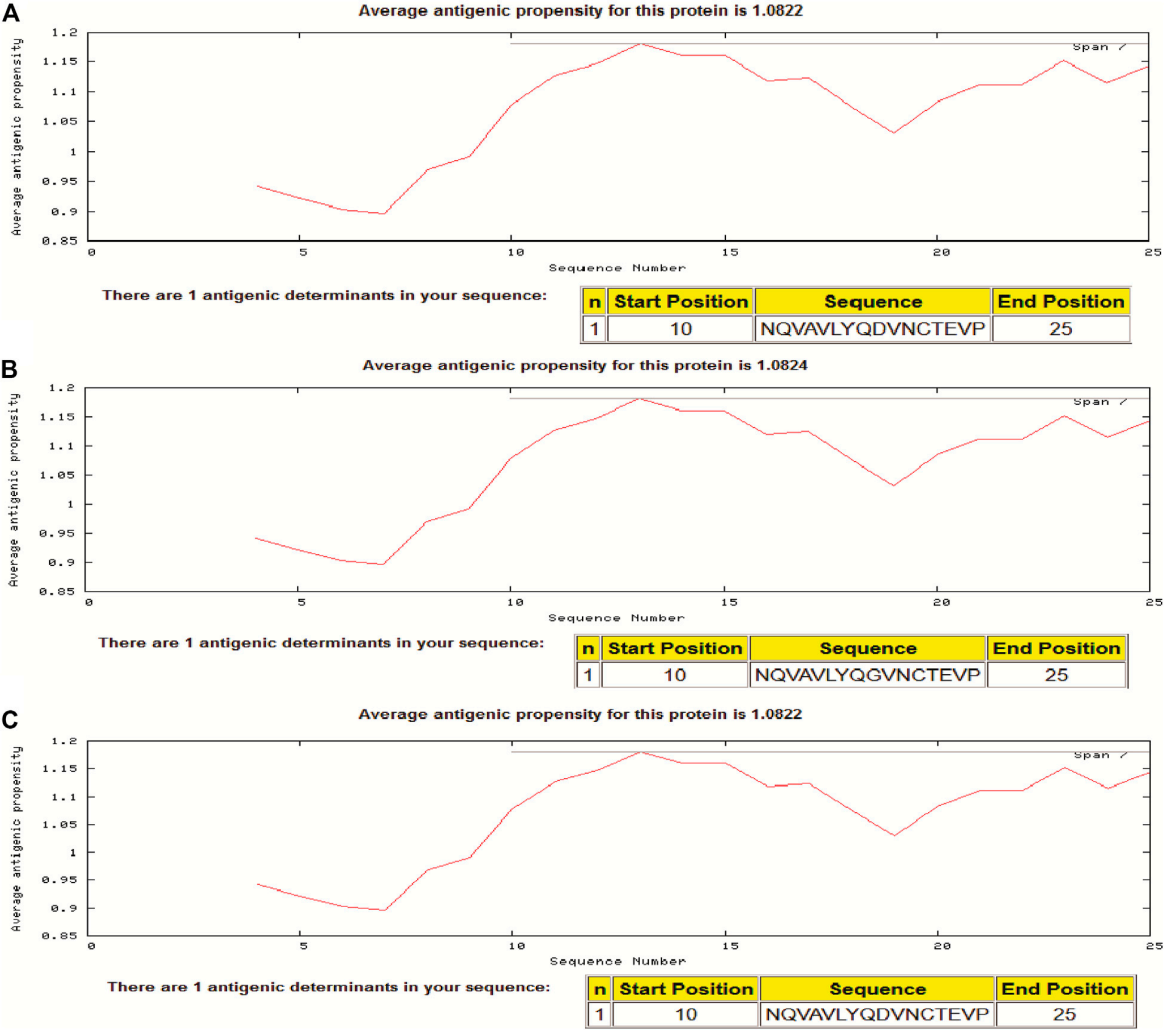
Antigenic propensity of two different SARS-2COV-2 sequences and Pangolin-CoV. **(A)** Pangolin-CoV, **(B)** SARS-CoV-2 (D614G) Variant, **(C)** Reference sequence at 614 position in spike glycoprotein sequence.

In the present study, we assessed the antigenic propensity of the peptides of the region S_597-625_ and the results showed that the variant having G at position number 614 is having a little higher antigenic propensity than the one having D at the same position. Isolate from Wuhan (i.e., reference having D614), a Pangolin CoV and a variant having G614 had antigenic propensity of 1.0822, 1.0822, and 1.0824, respectively. In each sequence of 29 residues, a peptide starting from position 10 to the 25th position comprised a single antigenic determinant (Figure 4).

## Discussion

A novel corona virus that emerged in December 2019 from Wuhan, China has resulted into a pandemic. In the structure of SARS-CoV-2 virus, spike protein is 1273 amino acid residue in length and forms a trimeric spike on the virion surface. There are many mutations observed in the amino acid composition of the spike protein but primary data shows that strains with S-D614G are more infectious and exhibit high transmission efficiency (Zhang et al., 2020). Regions between amino acid 614 and 621 of SARS-CoV-2 spike proteins were also identified as a B cell epitope by different methods and D614G may affect the antigenicity of this region (Kim et al., 2020). However, there is still much scope to understand how D614G affects antigenic properties of S protein; whether elastase-2 inhibitors and convalescent serum samples of patients can block infection of D614G variant remains unclear.

In the current study, we observed that D614G is highly prevalent mutation in the spike protein of genomes from COVID-19 patients of the Gujarat region. D614 is conserved in the reference sequence from Wuhan and a sequence from Guangzhou, while SARS-CoV-2 genome sequences from Gujarat, India showed a very high frequency of this mutation. In addition, it is concurrently seen with other mutations like P681R, L452R, T19R, E156G, T478K. These had >50% of occurrence in Gujarat whereas at the global level only D614G showed very high occurrence (98.53%) of all sequences and the rest of these formerly mentioned mutations had a frequency of 35–40% globally, lower than that in Gujarat. The observation that the P681R was the second most prevalent mutation reveals the recent high dominance of the Delta variant in the region. Additionally, spike D614G was accompanied by high occurrence of the nsp12 P4715L mutation, and this duo variant which is linked to pathogenicity was observed to be not linked positively to fatality rates in Africa (Lamptey et al., 2021). Such duo variants need further attention to assess host-based region-specific response.

Considering the high occurrence of D614G in spike protein of SARS-CoV-2, several groups have assessed the antigenic peptides and it is reported that the peptide S_597-625_ is one of the major immuno-dominant in humans (Wang et al., 2016; Kim et al., 2020). Antigenic propensity analysis showed that variant spike protein- G_614_ is having a little higher antigenic propensity to the D_614._ This observation may be one of the reasons for no change in the death rate despite the high spread of the variant. Further studies on spike protein epitopes may provide insights on the potential efficacy of many of the vaccines which may be designed based on the D614 sequence.

Earlier reports have also showed that D614G increases the efficiency of cellular entry for the virus across a broad range of human cell types, including cells from lung, liver, and colon (Daniloski et al., 2021). They also observed that spike-G_614_ is more resistant to proteolytic cleavage during the production of the protein in the host cell.

Seeing the rise in the cases with D614G mutation and its enhanced transmission, the D614G attracts significant consideration by researchers and healthcare field fellows. In the present work, we attempt to report the mutation analysis of spike protein and the antigenic propensity of D614G mutation in the spike protein of the viral isolates from the Gujarat region.

## Data Availability

The original contributions of SARS-CoV-2 genome sequences (n = 2439) presented in the study are publicly available. This data can be found here: https://www.gisaid.org/, the corresponding accession numbers are available from https://covid.gbrc.res.in/ (Sr. No. 1-2439).

## Supplementary Material

Supplementary Figure S1 Phylogenomic distribution of D and G variants **(A)** upto the studied genomes viz., July 2020 and **(B)** scenario down to the recent timeline viz., September 2021.

Supplementary Figure S2 Non synonymous mutations observed in the spike glycoprotein sequence of analysed genomes from Gujarat (*n* = 315) compared to the globally submitted genome sequences of SARS-CoV-2 compared to Wuhan reference by EpiCoVTM on GISAID platform as on June 30, 2020.
