# Supplementary figures and images for: Evolutionary and Antigenic Profiling of the Tendentious D614G Mutation of SARS-CoV-2 in Gujarat, India
Source: Front Genet. 2021 Nov 11;12:764927. doi: 10.3389/fgene.2021.764927 (PMC8632030; doi:10.3389/fgene.2021.764927)

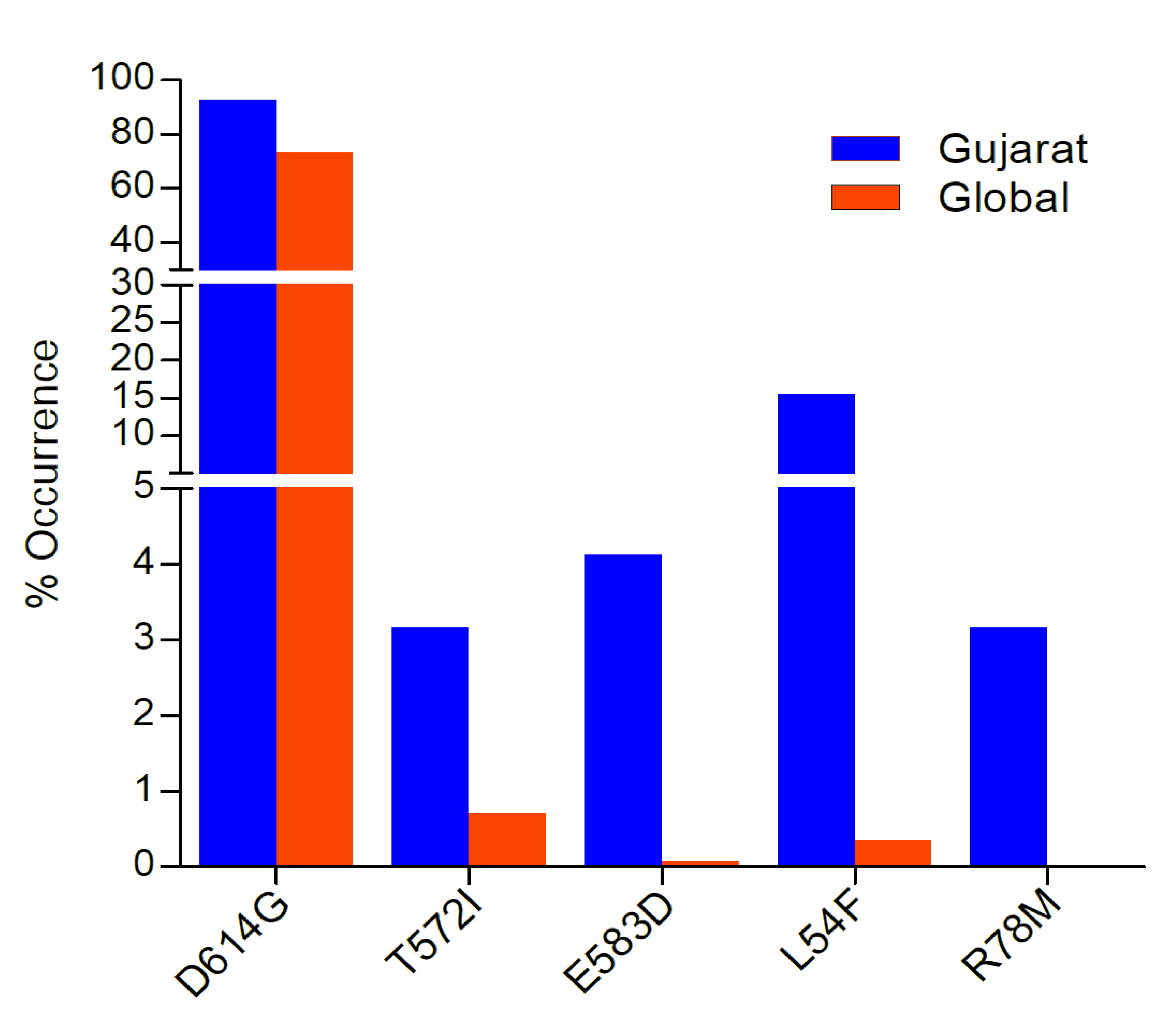

Supplement: Supplementary file 1 [file Image2.TIF]
